# Anti-Transforming Growth Factor ß Antibody Treatment Rescues Bone Loss and Prevents Breast Cancer Metastasis to Bone

**DOI:** 10.1371/journal.pone.0027090

**Published:** 2011-11-11

**Authors:** Swati Biswas, Jeffry S. Nyman, JoAnn Alvarez, Anwesa Chakrabarti, Austin Ayres, Julie Sterling, James Edwards, Tapasi Rana, Rachelle Johnson, Daniel S. Perrien, Scott Lonning, Yu Shyr, Lynn M. Matrisian, Gregory R. Mundy

**Affiliations:** 1 Department of Radiation Oncology, Vanderbilt University School of Medicine, Nashville, Tennessee, United States of America; 2 Center for Bone Biology, Vanderbilt University School of Medicine, Nashville, Tennessee, United States of America; 3 Department of Orthopedics, Vanderbilt University School of Medicine, Nashville, Tennessee, United States of America; 4 Vanderbilt University Institute of Imaging Science, Vanderbilt University School of Medicine, Nashville, Tennessee, United States of America; 5 Department of Cancer Biology, Vanderbilt University School of Medicine, Nashville, Tennessee, United States of America; 6 Department of Biostatistics, Vanderbilt University School of Medicine, Nashville, Tennessee, United States of America; 7 Genzyme Corporation, Framingham, Massachusetts, United States of America; Carl-Gustav Carus Technical University-Dresden, Germany

## Abstract

Breast cancer often metastasizes to bone causing osteolytic bone resorption which releases active TGFβ. Because TGFβ favors progression of breast cancer metastasis to bone, we hypothesized that treatment using anti-TGFβ antibody may reduce tumor burden and rescue tumor-associated bone loss in metastatic breast cancer. In this study we have tested the efficacy of an anti-TGFβ antibody 1D11 preventing breast cancer bone metastasis. We have used two preclinical breast cancer bone metastasis models, in which either human breast cancer cells or murine mammary tumor cells were injected in host mice via left cardiac ventricle. Using several *in vivo*, *in vitro* and *ex vivo* assays, we have demonstrated that anti-TGFβ antibody treatment have significantly reduced tumor burden in the bone along with a statistically significant threefold reduction in osteolytic lesion number and tenfold reduction in osteolytic lesion area. A decrease in osteoclast numbers (p = 0.027) *in vivo* and osteoclastogenesis *ex vivo* were also observed. Most importantly, in tumor-bearing mice, anti-TGFβ treatment resulted in a twofold increase in bone volume (p<0.01). In addition, treatment with anti-TGFβ antibody increased the mineral-to-collagen ratio *in vivo*, a reflection of improved tissue level properties. Moreover, anti-TGFβ antibody directly increased mineralized matrix formation in calverial osteoblast (p = 0.005), suggesting a direct beneficial role of anti-TGFβ antibody treatment on osteoblasts. Data presented here demonstrate that anti-TGFβ treatment may offer a novel therapeutic option for tumor-induced bone disease and has the dual potential for simultaneously decreasing tumor burden and rescue bone loss in breast cancer to bone metastases. This approach of intervention has the potential to reduce skeletal related events (SREs) in breast cancer survivors.

## Introduction

Breast cancer remains the second leading cause of cancer-related death among women, although in recent years there has been significant advancement in terms of treatment and prevention. This disease poses a serious threat due to the high incidence of metastasis to other organs, such as lung and bone. More than 75% of patients with breast cancer develop osteolytic bone metastasis, leading to tremendous bone loss [1], [2], [3], [4] and resulting in a significant burden on health care cost and poor quality of life for patients. Although currently used anti-resorptive therapies, such as bisphosphonates and denosumab, are successful in reducing further osteolysis, they cannot improve the existing damage in the residual bone [5], [6], [7], [8], [9], [10], [11]. Therefore, the surviving population remains prone to a high risk of skeletal-related events (SREs), such as pathological fracture, spinal cord compression, bone pain and hypocalcaemia [12], [13]. To address this issue, new therapeutic approaches to rescue cancer-induced bone loss are urgently required [14], [15].

Therapeutic approaches involving anti-TGFβ present an obvious choice in the rescue of cancer-induced bone loss for several reasons. Bone is the largest reservoir of TGFβ in the body and one of the major osteogenic cytokines. Both bone mass and bone matrix properties are regulated by TGFβ [16] and genetic manipulation of this pathway has been shown to affect bone mass in several murine models [17], [18], [19], [20]. Normal bone remodeling requires a balance between bone resorption and bone formation. However, in cancer-induced bone disease normal remodeling is interrupted. During the progression of osteolytic breast cancers, an increase in the osteoclastic bone resorption takes place. As a result, an excess of active TGFβ is secreted in the bone microenvironment, which in turn mediates a cascade of events that favor the vicious cycle of bone metastasis [21]. Although TGFβ is growth inhibitory for normal epithelial cells, it plays a favorable role in late tumor progression [22]. It has been demonstrated that active TGFβ signaling is needed for the establishment of bone metastasis [23]. This is in agreement with a study reporting higher plasma levels of TGFβ associated with poor prognosis in breast cancer patients [24]. Upon reaching the bone microenvironment, tumor cells are exposed to several growth factors including TGFβ which leads to upregulation of Gli2, a hedgehog family transcription factor. In osteolytic breast cancer cells, Gli2 has been shown to regulate the expression of parathyroid hormone related protein (PTHrP), a major osteolytic factor [25]. Intact TGFβ signaling in the breast cancer cells is necessary for the PTHrP secretion, suggesting a direct mechanistic link between TGFβ and tumor- induced osteolytic bone destruction [23]. In addition to promoting the growth of cancer cells in bone, TGFβ increases osteoclast differentiation [26], [27] and suppresses osteoblast differentiation [28]. All of these events contribute to the accelerated bone destruction in the tumor-infested bone, in a TGFβ-dependent manner. Increased TGFβ production in mice has been implicated in bone fragility and osteoporosis [18], suggesting that blockade of excess TGFβ may rescue bone loss. Therefore, anti-TGFβ antibody seems a logical approach to rescue bone loss.

Several attempts have been made to develop anti-cancer therapies involving an anti-TGFβ approach. Despite the predictable side effects, a number of anti-TGFβ compounds have been shown to inhibit primary and metastatic cancer and are in preclinical or clinical trials [29]. In a mouse model of bone cancer, blockade of TGFβ signaling in breast cancer cells has been shown to inhibit breast cancer to bone metastasis [23]. Yang et al. reported that lifelong treatment with a soluble TGFβ receptor II protects mice against metastasis [30]. A study using 4T1 mouse mammary cancer cells indicated that blocking TGFβ signaling systemically reduces metastatic events [31]. Small-molecule inhibitors of transforming growth factor β receptor I (TβR1) have been shown to reduce tumor burden in preclinical models of breast cancer bone metastasis and pulmonary metastasis [32]. Mohammad et al have recently shown that a small molecule inhibitor of TGFβ was able to inhibit melanoma bone disease in a preclinical model [33]. Whether these approaches also improve breast cancer-induced bone loss has not yet been reported.

Recently, a small molecule inhibitor of TβR1 kinase was shown to have anabolic and anti-catabolic effects on normal bone formation [34]. In addition, our group recently reported that the anti-TGFβ antibody has the potential to increase bone volume in normal mice [35]. These results prompted us to test the efficacy of anti-TGFβ antibody in preventing cancer-induced bone disease.

To investigate the effect of anti-TGFβ antibody on both tumor burden and bone loss, we obtained a pan-TGFβ antibody from Genzyme Corporation that blocks all three isoforms of TGFβ. Our *in vivo* results show that an anti-TGFβ antibody (1D11) significantly increased bone mineral density (BMD), trabecular thickness and bone volume, along with significant reduction in tumor burden and osteolytic bone damage in preclinical breast cancer bone metastasis models using both human and murine breast cancer cell lines. *In vitro*, 1D11 was able to block TGFβ induced expression of both Gli2 and PTHrP, which provides a mechanistic explanation of reduced tumor burden in our model. To our knowledge, this is the first demonstration of dual efficacy of an anti-TGFβ antibody to both inhibit tumor burden and rescue bone loss in a breast cancer to bone metastasis model [33].

## Materials and Methods

### Animals

All procedures were performed with the approval of the Vanderbilt University Institutional Animal Care and Use Committee and in accordance with Federal guidelines. For all *in vivo* experiments, 4- to 5-week-old female athymic nude mice (for MDA-MB-231 human breast cancer cells) or Balb/C mice (for 4T1 mouse mammary tumor cells) were used.

### Study design

Both the anti-TGFβ (1D11) and control antibody (13C4), directed against Shigella toxin, were obtained from Genzyme Corporation, MA. To test the efficacy of anti-TGFβ antibody 1D11 in the inhibition of bone metastases, we used preclinical models of breast cancer to bone metastases. Mice were inoculated with breast tumor cells into the left cardiac ventricle and were treated with either anti-TGFβ antibody (1D11, 10 mg/kg body weight) or control antibody (13C4, 10 mg/kg body weight), starting either one day after tumor cell inoculation (the adjuvant, or metastasis prevention regimen) or 2 weeks after tumor cell inoculation (the established metastasis regimen); in both regimens, treatment frequency was 3 days per week and continued until 4 weeks after tumor cell inoculation. Any mice showing the sign of distress before this period was sacrificed immediately. 1D11 is a murine monoclonal antibody which is able to neutralize all three isoforms of TGFβ *in vitro* [36] and *in vivo* [36], [37], [38]. This antibody only recognizes the active form of the cytokine. The vehicle used for preparing the antibodies showed no significant difference in the tumor burden in comparison to the control-antibody-treated group during initial experiments and was therefore excluded from these studies (communication with Genzyme Corporation). The outcome measures included quantification of osteolytic bone destruction using X-ray and histology. Additionally, trabecular bone volume and architecture were measured using microCT. Bone quality parameters were measured using Confocal Raman spectroscopy. Tumor burden and osteoclast numbers were quantified by means of histology.

### Cell culture

The human cancer cell line MDA-MB-231 was obtained from ATCC (American Type Culture Collection), and a bone metastatic variant generated and reported previously by our group [39] was used for all *in vitro* and *in viv*o studies. The murine mammary cell line 4T1 had previously been obtained from another investigator [40] and used in a cardiac injection model within our group [41]. Both cell lines were maintained in DMEM (Invitrogen, Carlsbad, CA) containing 10% Fetal Bovine Serum (FBS: Hyclone Laboratories, Logan, UT) and 1% penicillin/streptomycin (Mediatech). Cells were cultured in a 37°C atmosphere of 5% CO_2_ and 95% O_2_ using standard tissue culture techniques.

### Intracardiac bone metastasis model

MDA-MB-231 or 4T1 cells were trypsinized, washed and then resuspended in ice-cold sterile PBS at a final concentration of 1×10^6^/ml. Four- to 5-week-old female nude (for MDA-MB-231cell injection) or Balb/C (for 4T1cell injection) mice were anesthetized using a ketamine/xylazine mixture. Mice were positioned ventral side up, and tumor cells were injected into the left cardiac ventricle using a percutaneous approach with a 27-gauge needle attached to a 1 ml syringe, as described previously [42], [23]. Correct injection position in the left ventricle was confirmed by the appearance of bright red blood at the hub of the needle in a pulsatile fashion. Each mouse received 1×10^5^ cells in a 100-µl volume (resuspended in PBS) which was injected slowly over 1 minute. For the adjuvant, or metastasis prevention regimen, mice were treated starting 1 day after tumor cell inoculation. For established metastasis protocol, mice were imaged until visible lesion detection (approximately two weeks) and treatment was started at that point and was continued for another two weeks. All mice were imaged weekly and sacrificed 4 weeks post-tumor inoculation. Any mice showing signs of distress prior to 4 weeks were sacrificed immediately.

### Radiographic analysis of bone lesions

Beginning 1 week after tumor cell inoculation, tumor-bearing animals were subjected to radiographic imaging. In brief, mice were sedated using ketamine/xylazine and placed in a prone position. X-ray images were then taken at 35 kVp for 8 s using a digital radiography system (Faxitron LX-60). Images were saved and lesion area and lesion numbers were evaluated using image analysis software (Metamorph, Molecular Devices, Inc.). Data presented are the average of lesion area and lesion numbers per mouse in each treatment group.

### Bone histology and histomorphometry

After sacrifice, hind limbs (tibiae and femora) from each mouse were harvested, fixed in 10% neutral-buffered formalin (Fisher Scientific) for 48 h and stored in 70% ethanol for further processing. Following microCT analysis, the tibiae and femora were decalcified in 10% EDTA for two weeks and embedded in paraffin using an automated tissue processor for histological analysis. Mid-sagittal sections (5 µm) of tibiae or femora were stained with hematoxylin, orange G (Sigma) and phloxine B (Sigma). Separate sections were also stained for TRAPC activity for visualization of osteoclasts. Histomorphometric analysis of tumor burden, osteoclast numbers and osteoblast numbers was conducted on digital micrographs (100×) using an image quantification software (Metamorph, Molecular Devices, Inc) software. Tumor burden, defined as area occupied by tumor within the medullary region, was calculated. Osteoclasts numbers per area of trabecular bone surface was measured in a blinded fashion in the TRAP stained mid section of long bones using Metamorph software.

### Quantitative microCT

MicroCT analysis was performed in the Vanderbilt University Institute of Small Animal Imaging. To assess the effect of anti-TGFβ treatment on the architecture and structure of bone in tumor bearing mice, long bones were used. Micro-computed X-ray tomography (MicroCT) was used to measure trabecular bone volume within the metaphysis of the tibia and trabecular bone volume, architecture and density in the metaphysis of the femur. The long axis of each specimen was aligned with the scanning axis. One hundred slices from the proximal tibia were scanned at a 12-µm resolution (µCT40 Scanco Medical, Switzerland). The region of interest was trabeculae within the proximal metaphysis of the tibia (0.24 to 1.20 mm) below the growth plate. Images were acquired using 55 kV, 114 µA, 300-ms integration, and 500 projections per 180° rotation. Contiguous cross sectional images of the entire metaphyseal region were acquired. Following reconstruction, the bone tissue was segmented from air or soft tissue using a threshold of 270 per thousand (or 438.7 mgHA/cm^3^), a Gaussian noise filter of 0.8 and support of 2. Standard architectural characteristics such as trabecular bone volume (BV/TV), trabecular thickness (Tb.Th*), trabecular number (Tb.N*), connectivity density (Conn.D) and mean volumetric density of the mineralized tissue (Tb.TMD or mBMD) were calculated using the Scanco evaluation software.

### Raman micro-Spectroscopy

To determine whether neutralizing TGFβ in the tumor-bone microenvironment affected composition at the tissue level, we collected Raman spectra from the cortex of the tibial metaphysis. Chemical bonds naturally absorb a small amount of energy from a laser photon as they vibrate (known as Raman scattering of light). In Raman spectroscopy, the spectrum of reflected light (shift in wave number) is collected after the laser excites the bonds in a tissue at a given wavelength, which for these purposes is near-infrared (785 nm). This spectrum then characterizes the physiochemical properties of the tissue. Using the confocal Raman microscope (Renishaw Inc., Ramanscope Mark III) with a spatial resolution of 2–5 µm and a spectral resolution of 1 cm^−1^, we acquired nine spectra from polished sections of embedded tibia (∼150 µm below growth plate) and quantified the intensities of key peaks related to mineral (*v*_1_ phosphate and Type-B carbonate) and collagen (proline ring). Thus, mineral-to-collagen ratio, type-B carbonate substitution and crystallinity were the averages of *v*_1_ phosphate/proline, v_1_ phosphate/carbonate, and the inverse of the full width at half the maximum of v_1_ phosphate per bone.

### Osteoclastogenesis assay

Mouse long bones were flushed with PBS, resuspended by pipeting, and strained through a cell strainer (BD Biosciences, 40 µM). Mononuclear cells were isolated from resuspended bone marrow using Histopaque 1077 (Sigma), following manufacturers' instructions. Cells were plated in alpha-MEM media supplemented with 10% fetal bovine serum, RANKL (100 ng/ml) and MCSF (30 ng/ml) to support osteoclast formation. Both reagents were obtained from R&D systems. Two treatment groups were used, one treated with isotype antibody (13C4, 25 ug/ml) and other treated with anti-TGFβ antibody (1D11, 25 ug/ml). TRAP staining was performed using Leukocyte Acid Phosphatese kit (Sigma) and number of osteoclasts per field was counted under microscope.

### Osteoblast differentiation assay

Primary cultures of calvarial osteoblasts were prepared using a modified sequential collagenase/trypsin digestion method [43]. Briefly, calvaria were removed from 3- to 4-day-old C57Black 6 mice, cleaned free from soft tissue, washed for 10 min with PBS containing 0.025% trypsin, and digested with type-IV Collagenase p (1 mg/ml; source: *Clostridium histolyticum*, Roche) and 0.025% trypsin for 30 min at 37°C in HBSS with gentle agitation. The procedure was repeated twice, with a 1-h digestion followed by a 30-min digestion using above mentioned concentration of collagenase p and trypsin. The cells from the second and third digestions were collected and centrifuged at 2500×g for 10 min. The supernatant was aspirated and discarded, and the pellet was resuspended in alpha-MEM containing 10% fetal bovine serum. The culture was kept undisturbed for at least 2 days. At confluence, cells were trypsinized using the standard procedure and plated in 24 well plates for osteoblast differentiation assay. Cells were cultured in alpha-MEM containing 2.5% fetal bovine serum for a further 3 weeks in the presence of 5 mM beta glycerophosphate (Sigma) and 100 ug/ml L-ascorbic acid (Sigma) either in the presence of isotype control antibody (13C4, 25 µg/ml) or anti-TGFβ antibody (1D11, 25 µg/ml). 6 wells were dedicated to each treatment group. Media containing ascorbic acid and beta glycerophosphate was changed every 2 days until mineralized nodules (approximately 15–28 days) were formed. Mineralized matrix formation was detected by means of Von Kossa staining and quantified using Metamorph image analysis software.

### Co-culture assay

*Ex vivo* co-culture assay was done using mouse calverial osteoblasts and adult mouse bone marrow mononuclear cells. Calverial osteoblasts were isolated from 3–4 days old pups following the method described previously [43] and cultured in 6 well tissue culture plates until confluent. After these cells were confluent, bone marrow mononuclear cells were isolated from normal mice and plated on top of the osteoblast layer. The co-culture system was treated with either control antibody (13C4, 25 µg/ml) or anti-TGFβ antibody (1D11, 25 µg/ml) every other day for 7–10 days. Cells were fixed and stained for assessment of mature osteoclasts formation using Leucocyte Acid Phosphatase kit (Sigma) according to manufacturer's instruction and mature osteoclasts (red) were scored using microscope.

### Quantitative real-time PCR

Total RNA was extracted using RNeasy Mini Kit (QIAgen) according to the manufacturer's instruction. cDNA was synthesized using SuperScript III First-Strand Synthesis System for RT-PCR (Invitrogen) and random hexamers from 2 µg of total RNA per manufacturer's instructions. cDNA (2 µg) was used for quantitative real-time PCR using the Real MasterMix (Eppendorf, Hamburg, Germany) and 0.5 µL of prepared cDNA per manufacturer's instructions. Real-time PCR was done in triplicate using the Real Plex Machine (Eppendorf) with the following cycling conditions: 95°C for 15 seconds, 58°C for 30 seconds, and 68°C for 30 seconds. Normalization was done using 18S as an internal control.

### Statistical Considerations

The data are presented using box plots showing the quartiles along with the raw data, plotted separately for each group and for each outcome. Wilcoxon rank-sum tests and Kruskal-Wallis tests were used to test the null hypotheses of no difference in the distribution of the outcomes among the treatment groups. All analyses were performed using R version 2.11.1. *In vivo* results presented are from the 4 week treatments; however, the two week treatment showed similar outcome.

## Results

### Anti-TGFβ antibody treatment reduces tumor burden in bone

**Figure 1:**
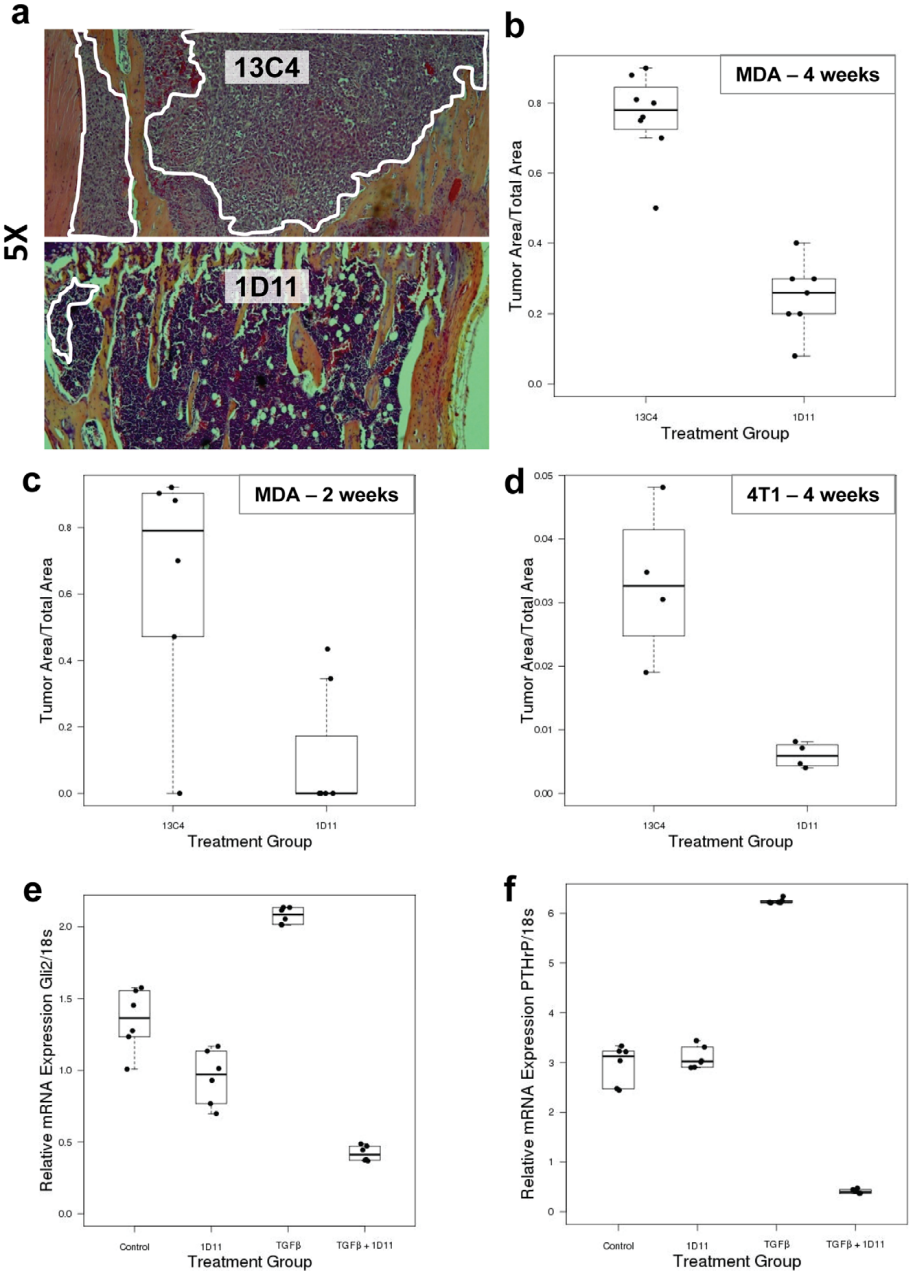
Anti-TGFβ antibody treatment decreases tumor burden in tumor-bearing mice. **Panel a:** Representative H&E sections (5×) of tibia from tumor-bearing mice treated with control antibody (13C4) or anti-TGFβ antibody (1D11). White line indicates the presence of tumor. **Panel b:** A boxplot of tumor burden in MDA-MB-231 tumor-bearing mice treated with either 13C4 (10 mg/kg) or 1D11(10 mg/kg) for 4 weeks, starting 1 day after tumor cell injection (N = at least 7) showing decrease in tumor burden. Wilcoxon rank-sum p-value = 0.001. Mean ± standard deviation = 13C4: 0.76±0.12, 1D11: 0.25±0.1. **Panel c:** Boxplots of tumor burden in MDA-MB-231 tumor bearing mice treated with either 13C4(10 mg/kg) or 1D11(10 mg/kg) starting two weeks after tumor cell injection and continued to be treated until the end of 4 weeks post tumor injection. Wilcoxon rank-sum p-value = 0.016. Mean ± standard deviation = 13C4:0.6461 ±0.3599, 1D11: 0.1114±0.1919. (N = at least 5). **Panel d:** Boxplot of tumor burden by group for the 4T1 tumor bearing mice (4T1 cells injected in Balb/c), treated 1 day post tumor cell injection and treated for 4 weeks shown decrease in tumor burden. Wilcoxon rank-sum p-value = 0.03. Mean ± standard deviation = 13C4: 0.0331±0.012, 1D11:0.006±0.002 (N = 4). **Panel e:** Decreased relative mRNA expression of Gli2 in MDA-MB-231 upon 1D11 treatment. Wilcoxon rank-sum p-value for TGFβ versus TGFβ+1D11 groups is 0.005. Mean ± standard deviation = TGFβ: 2.08±0.06, TGFβ + 1D11: 0.42±0.05. Results presented here are representative of at least two independent experiments. **Panel f:** Decreased relative mRNA expression of PTHrP in MDA-MB-231 upon treatment with 1D11. Wilcoxon rank-sum p-value for TGFβ versus TGFβ + 1D11 groups is 0.005. Mean ± standard deviation = TGFβ: 6.24±0.05, TGFβ + 1D11: 0.41±0.04. Results presented here are representative of at least two independent experiments.

Using two preclinical mouse models of breast cancer to bone metastases, we have assessed the efficacy of the anti-TGFβ antibody 1D11 in reducing tumor burden. Female nude mice (4 weeks old) were inoculated with MDA-MB-231 cells via the intracardiac route. Mice were treated with either control antibody (13C4) or anti-TGFβ antibody (1D11), either from one day after tumor cell inoculation (the adjuvant, or metastasis prevention regimen) or 2 weeks after tumor cell inoculation (the established metastases regimen), as described in the Materials and methods section. Following 4-weeks of treatment, anti-TGFβ treatment significantly reduced the tumor burden in the long bones (*p* value = 0.001; Figure 1b) and only microscopic small foci of tumor cells were observed in most mice treated with 1D11 (Figure 1a, white line indicates area occupied by tumor). Following 2-weeks treatment (established metastases protocol), a similar but less dramatic effect was observed (*p* value = 0.016; Figure 1c).

To test whether this treatment was effective in other bone metastases models, female Balb/C mice was inoculated with 4T1 murine mammary breast cancer cells and mice were treated one day after tumor cell inoculation and continued to be treated for 4 weeks. Tumor burden was significantly reduced in mice treated with anti-TGFβ antibody compared to the isotype control group (*p* = 0.03, Figure 1d).

### Anti-TGFβ antibody reduced PTHrP and Gli2 expression in breast cancer cells

It has been reported that TGFβ can upregulate the expression of Gli2, a hedgehog signaling molecule which is one of the driving factors of osteolytic bone metastasis, we have tested whether treatment with 1D11 might suppress Gli2 expression. As anticipated, TGFβ-induced expression of Gli2 was decreased when MDA-MB-231 tumor cells were treated with 1D11 (Wilcoxon rank-sum p-value for TGFβ versus TGFβ and 1D11 is 0.005) (Figure 1e). This might be one of the mechanisms by which anti-TGFβ antibody 1D11 inhibited osteolytic bone damage in our model. Gli2 is also known to increase the secretion of parathyroid hormone-related protein (PTHrP), another major osteolytic factors, in a TGFβ dependent process. Inhibition of PTHrP can prevent tumor induced bone destruction, therefore, we tested whether by neutralizing excess TGFβ, 1D11 may also decrease PTHrP expression in the tumor cells. Using real time PCR, we found that 1D11 significantly reduced TGFβ-induced expression of PTHrP in the MDA-MB-231 cells (Wilcoxon rank-sum p-value for TGFβ versus TGFβ and 1D11 is 0.005) (Figure 1f).

### Anti-TGFβ antibody treatment reduces osteolytic lesions in MDA-MB-231 cardiac injection model

**Figure 2:**
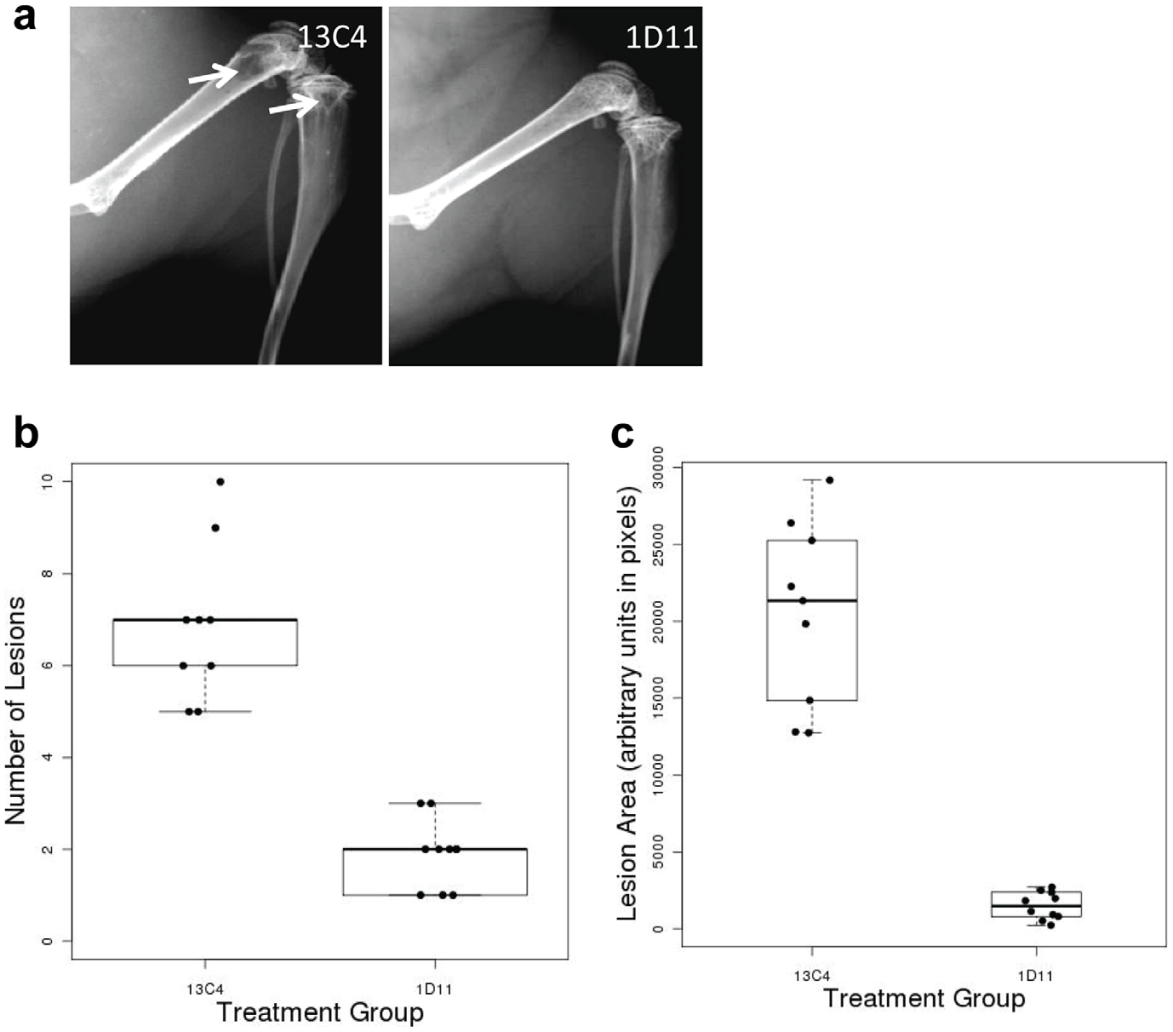
Anti-TGFβ antibody reduces osteolytic lesions in MDA-MB-231 breast cancer bone metastasis cardiac injection model. Mice were inoculated with MDA-MB-231 human breast cancer cells in the left cardiac ventricle and were treated with either isotype control (13C4, 10 mg/kg) or anti-TGFβ antibody (1D11, 10 mg/kg) for 4 weeks, starting from 1 day post tumor cell injection. At the end of the experiment, whole body X-ray images of mice from both control and anti-TGFβ antibody treated group were taken and osteolytic lesion area and osteolytic lesion counts were analyzed using image analysis software (Metamorph, Molecular Device). **Panel a:** Representative X-ray images of osteolytic bone lesions in the hind leg of mice treated for 4 weeks either with control antibody (13C4, left panel) or anti-TGFβ antibody (1D11, right panel). White arrows indicate presence of osteolytic lesions. **Panel b:** A boxplot representing the average lesion counts in mice inoculated with MDA-MB-231 cells in the left cardiac ventricle, treated with either control antibody (13C4, 10 mg/kg) or anti-TGFβ antibody (1D11, 10 mg/kg) for 4 weeks, starting 1 day after tumor cell injection shows decrease in lesion numbers after anti-TGFβ treatment (6.9±1.7 for control and 1.9±0.7 for 1D11; Wilcoxon rank-sum p-value = <.001, N = 9). **Panel c:** A boxplot representing the lesion area from the same experiment shows decrease in the lesion area after anti-TGFβ treatment (20520±6000 for control and 1497±888 for 1D11; Wilcoxon rank-sum p-value = <.001, N = at least 9). Lesion areas were measured using arbitrary pixel unit.

Advanced metastatic breast cancer in patients leads to severe osteolytic damage. MDA-MB-231 cells injected via the left cardiac ventricle of female nude mice give rise to comparable metastatic lesions, which can be quantified using X-ray image analysis. Radiographic image analysis in MDA-MB-231 tumor-bearing mice treated with either control antibody (13C4, 10 mg/kg, Figure 2a, left panel, arrow indicating osteolytic damage) or anti-TGFβ antibody (1D11, 10 mg/kg, Figure 2a, right panel) has indicated that the average number of osteolytic lesions was reduced by more than three-fold (Figure 2b; p<0.001) and the average lesion area was reduced ten-fold in the anti-TGFβ treatment group when compared with control (Figure 2c; p<0.001).

### Anti-TGFβ antibody treatment reduces osteoclast numbers in tumor-bearing mice

**Figure 3:**
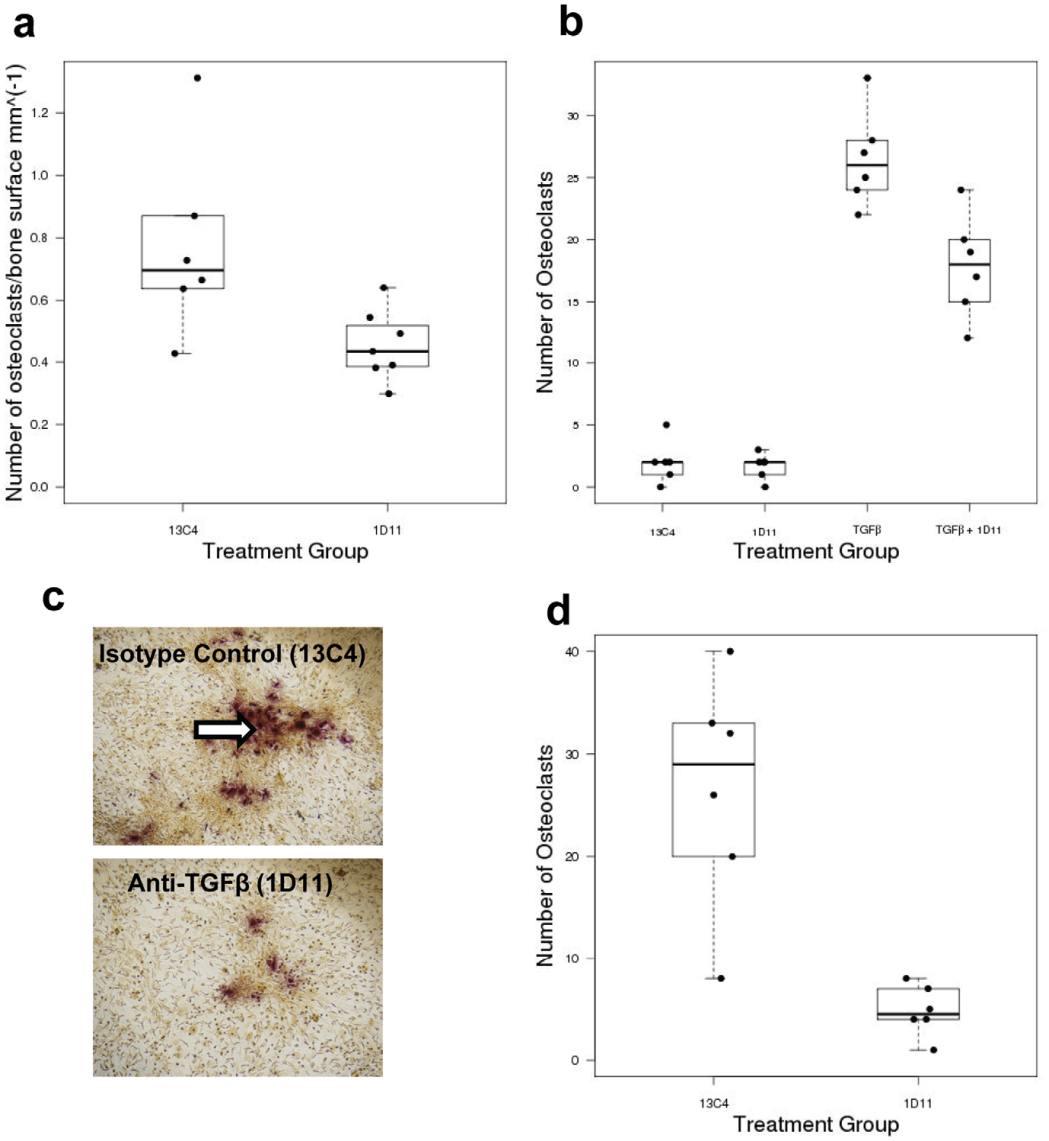
Anti-TGFβ antibody decreased osteoclast numbers and *in vitro* osteoclastogenesis. **Panel a:** Boxplot of number of TRAP positive osteoclasts per millimeter of bone surface in MBA-MB-231 tumor-bearing tibiae, showing significantly decreased osteoclasts upon 1D11 treatment, compared to 13C4. Wilcoxon rank-sum p-value = 0.027. Mean ± standard deviation = 13C4: 0.7733±0.3002, 1D11: 0.4539±0.1141. N = at least 6). **Panel b:** To assess the effect of anti-TGFβ treatment directly on osteoclast population, an *ex vivo* osteoclastogenesis assay was performed using bone marrow mononuclear cells. Bone marrow mononuclear cells were isolated and cultured in presence of 13C4 (control antibody), 1D11(anti-TGFβ antibody), TGFβ+13C4 or TGFβ+1D11 in presence of both RANKL and MCSF. Both 13C4 and 1D11 was used at a concentration of 25 µg/ml. TGFβ as used at a concentration of 5 ng/ml. Osteoclasts were stained using using a Leucocyte acid phosphatase (TRAP) kit as per manufacturer's instruction (Sigma-Aldrich) and TRAP positive cells (reddish brown) were counted under microscope. Boxplots of number of osteoclasts by group for osteoclastogenesis assay show that treatment with 1D11 significantly reduced TGFβ-mediated osteoclast formation. Wilcoxon rank-sum p-value for TGFβ and TGFβ + 1D11 groups is 0.01. Mean ± standard deviation = TGFβ: 26.5±3.83, TGFβ + 1D11: 17.83±4.17. Data presented here is representative of two independent experiments. **Panel c:** To assess the effect of anti-TGFβ antibody on osteoblast-mediated osteoclastogenesis, bone marrow mononuclear cells were cultured on a layer of primary mouse calverial osteoblasts in the presence of either control antibody (13C4) or the anti-TGFβ antibody (1D11). After 7–10 days, TRAP staining was performed to identify mature osteoclasts (indicated by arrow). **Panel d:** Osteoblast mediated osteoclastogenesis increases significantly upon 1D11 treatment compared to control, Wilcoxon rank-sum p-value = 0.006. Mean ± standard deviation = 13C4: 26.5±11.31, 1D11: 4.83±2.48. Data presented here is representative of two independent experiments.

Increased bone resorption in the tumor-bearing bone has been associated with increased osteoclast numbers. Since, X-ray imaging has revealed that treatment with anti-TGFβ antibody resulted in fewer osteolytic lesions and overall smaller osteolytic area, we have anticipated that, anti-TGFβ treatment may reduce number of osteoclasts *in vivo*. Mouse long bones from both control antibody and anti-TGFβ antibody treatment groups were stained with TRAP stain. As anticipated, histological analysis revealed that the number of TRAP-positive osteoclasts per millimeter of bone surface was significantly lower in the group treated with anti-TGFβ antibody (1D11) compared to the isotype control (13C4) (Figure 3a; p = 0.027).

Bone marrow microenvironment is a complex multicellular system. The differentiation of osteoclasts is also regulated by osteoblasts. Therefore, fewer osteoclasts *in vivo* may be a direct effect of the treatment on the osteoblast precursor cells (bone marrow mononuclear cells) or mediated via osteoblasts.

In an attempt to test the direct effect of anti-TGFβ treatment on osteoclastogenesis, mononuclear cells were isolated from mouse bone marrow and subjected to osteoclast differentiation, either in the presence control antibody (13C4) or anti-TGFβ antibody (1D11) (Figure 3b). As anticipated, TGFβ significantly increases the number of TRAP-positive osteoclasts and treatment with 1D11 significantly reduced TGFβ-mediated osteoclast formation (*p* = 0.024, compared between TGFβ versus TGFβ and 1D11 treatment).

By secreting both OPG and RANKL, osteoblasts maintain the homeostasis of osteoclasts in bone microenvironment [44], [45], [46], [47], [48]. Since TGFβ has also been reported to alter the RANKL/OPG ratio, we asked whether the TGFβ neutralizing antibody interfered with osteoblast-induced osteoclastogenesis. Mouse calvarial osteoblasts and mouse bone marrow mononuclear cells were co-cultured in the presence of the control antibody or anti-TGFβ antibody (3c, representative images. Arrow indicates presence of TRAP positive osteoclasts). The anti-TGFβ treatment resulted in almost fivefold reduction the number of osteoclasts (Figure 3d, Wilcoxon rank-sum p value = 0.006). The result of the *ex vivo* assay are in agreement with the *in vivo* data and reinforce the notion that TGFβ plays a critical role in tumor-induced bone resorption at least in part through the induction of osteoclastogenesis.

### Anti-TGFβ antibody treatment increases bone volume and improves bone architecture in breast cancer to bone metastasis

**Figure 4:**
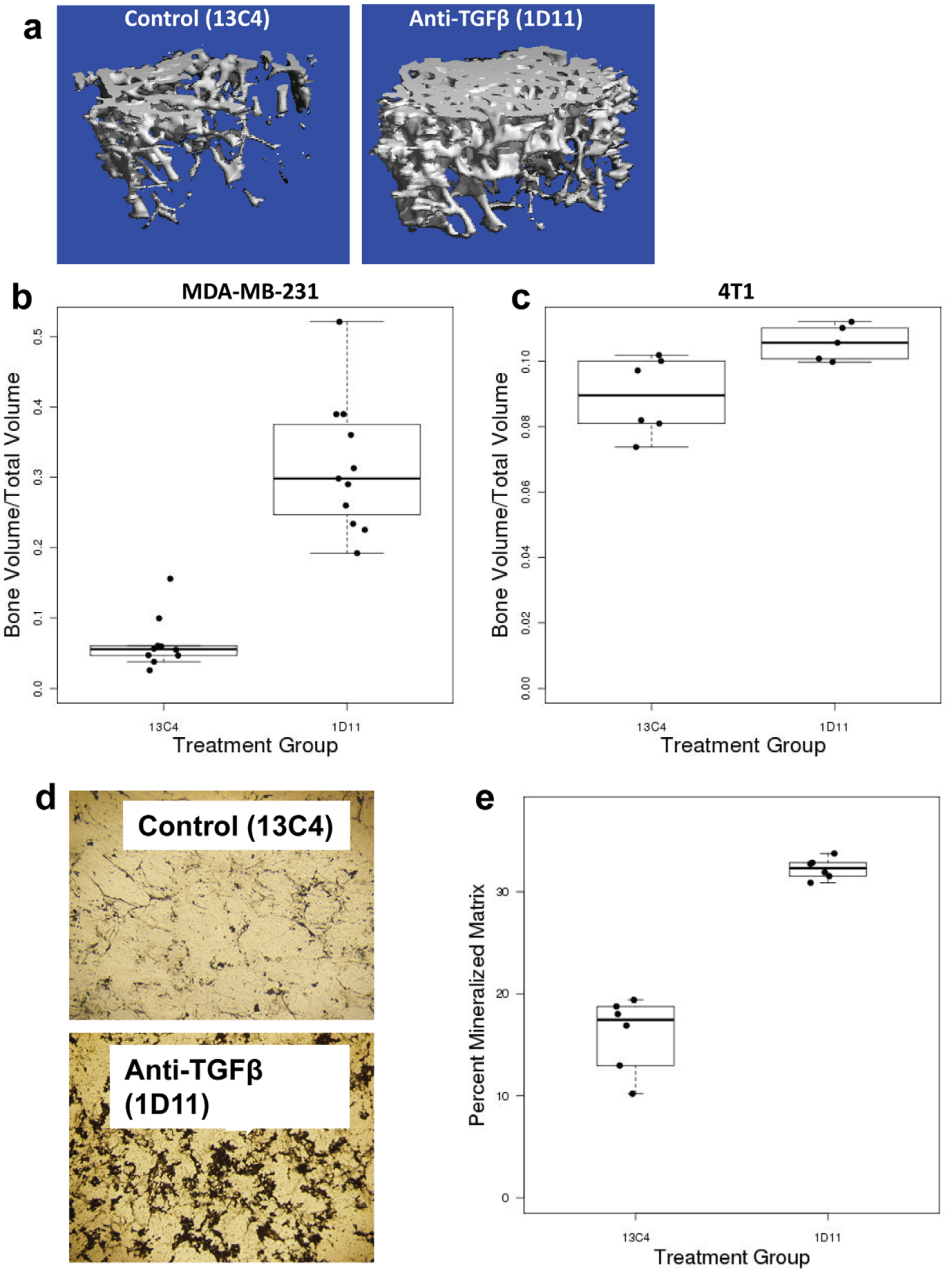
Anti-TGFβ antibody increases bone volume in tumor bearing mice. MDA-MB-231 cells were injected via intra-cardiac route in 4 week old female nude mice and 4T1 cells were injected in 4–5 week old female Balb/C mice. Mice were treated either with control antibody (13C4, 10 mg/kg) or anti-TGFβ antibody (1D11, 10 mg/kg) for 4 weeks, starting 1 day after tumor cell inoculation. Trabecular bone volume in the tibial metaphysis of tumor-bearing mice was analyzed by microCT. **Panel a:** Representative three dimensional reconstitutions of microCT images from both 13C4 and 1D11 treated groups from mice injected with MDA-MB-231 cells. **Panel b:** Boxplots of average BV/TV (bone volume/total volume) by group for the MDA-M**B**-231 tumor- bearing mice show significant increase in bone mass after treatment with anti-TGFβ antibody. Wilcoxon rank-sum p-value = <0.001. Mean ± standard deviation = 13C4: 0.06±0.04, 1D11: 0.32±0.09. N = at least 10. **Panel c.** Boxplots of average BV/TV (bone volume/total volume) by group for the 4T1 tumor- bearing mice show a significant increase in bone mass as a result of treatment with 1D11 as measured by BV/TV. Wilcoxon rank-sum p-value = 0.036. Mean ± standard deviation = 13C4: 0.09±0.01, 1D11: 0.11±0.01, N = at least 5. **Panel d:** Mouse calverial osteoblasts were isolated and cultured for 7–10 days as described in the Materials and Methods, either in presence of anti-TGFβ antibody (1D11) or isotype control (13C4) and mineralized matrix formation was measured using Von Kossa staining as a surrogate for osteoblast differentiation. **Panel e:** Boxplot analysis reveals treatment with anti-TGFβ antibody (1D11) significantly increased percent areas of mineralized matrix. Images were taken from representative fields and quantified using Metamorph software. Wilcoxon rank-sum p-value = 0.005. Mean ± standard deviation = 13C4: 16±3.7, 1D11: 32.3±1. Data presented here is representative of two independent experiments.

We have used either MDA-MB-231 cells and injected those in the left ventricle of nude mice or 4T1 cells and injected those in Balb/C mice for assessing the efficacy of anti-TGFβ treatment in cancer induced bone disease. Representative 3D images of mice tibiae from both 13C4 (left panel) and 1D11 (right panel) treatment groups are shown in figure 4a, from the experiment where MDA-MB-231 cells were injected in nude mice. MicroCT analysis of tibia from mice bearing MDA-MB-231 human breast cancer cells in bone following intra-cardiac injection demonstrated that a 4-week treatment with anti-TGFβ antibody 1D11 resulted in an approximately 5-fold increase in the overall bone volume when compared with the isotype control 13C4 treated group (Figure 4b; p<0.001). A similar but less dramatic effect was observed with murine 4T1 mammary cancer cells injected into syngeneic Balb/C mice via intra-cardiac route (Figure 4c; p<0.036). Further analysis using quantitative microCT showed that treatment with 1D11 resulted in a greater number of trabeculae, in thicker trabeculae, and in higher connectivity density (lack of fenestrations) of the trabecular bone, suggesting that suppression of TGFβ improves trabecular architecture in the presence of a tumor (Table 1). However, bone mineral density (mBMD or Tb.TMD) of the trabeculae was unchanged.

**Table 1:** Anti-TGFβ antibody improves trabecular architecture in tumor bearing mice tibia and femur.

Tibia	N	13C4 (N = 10)	1D11 (N = 11)	p value
Bone Volume/Total Volume	21	0.064±0.037	0.316±0.095	p<0.001
ConnD mm^−3^	21	40.414±42.618	277.836±73.712	p<0.001
SMI	21	2.788±0.355	0.701±0.946	p<0.001
Tb.N mm^−1^	21	2.699±0.69	6.553±1.1	p<0.001
Tb.Th mm	21	0.044±0.004	0.058±0.007	p<0.001
Tb.Sp mm	21	0.396±0.098	0.146±0.03	p<0.001
Tissue MineralDensity (mgHA/cm3)	21	1031.672±43.286	1008.325±24.358	p = 0.091

Femur	N	13C4 (N = 9)	1D11 (N = 6)	p value
Bone Volume/Total Volume	15	0.073±0.043	0.247±0.08	p = 0.002
ConnD mm-3	15	60.685±43.046	207.442±83.689	p = 0.003
SMI	15	2.778±0.455	1.364±0.645	p = 0.002
Tb.N mm-1	15	2.99±0.756	5.371±0.939	p = 0.002
Tb.Th mm	15	0.046±0.004	0.057±0.004	p = 0.002
Tb.Sp mm	15	0.353±0.069	0.187±0.035	p = 0.002
Tissue Mineral Density (mg HA/cm3)	15	977.687±40.562	985.161±20.43	p = 0.814

MicroCT analysis of the tibiae from MDA-MB-231tumor-bearing mice treated for 4 weeks, starting one day after tumor cell inoculation, revealed that suppression of TGFβ by the antibody 1D11 increased trabecular bone volume through increases in trabecular number, and this improved the connectivity of the trabeculae (lack of fenestrations), compared to isotype control. Wilcoxon rank-sum test was used for this analysis. Means and standard deviations by group for the MDA-MB-231 four week data with p-values from Wilcoxon rank-sum tests. Quantitative analysis of microCT data from MDA-MB-231 tumor-bearing mice treated with 13C4 or 1D11 for weeks. Trabecular bone volume (BV/TV), trabecular thickness (Tb.Th*), trabecular number (Tb.N*), and connectivity density (Conn.D), and mean volumetric density of the mineralized tissue (Tb.TMD) were calculated using the Scanco evaluation software.

### Anti-TGFβ antibody increases osteoblast differentiation *in vitro*

To investigate whether increased bone volume and improved architecture observed in anti-TGFβ antibody-treated animals are a reflection of increased osteoblast differentiation, an *ex vivo* osteoblast differentiation assay was performed. Mouse calverial osteoblasts were isolated and cultured in presence of either isotype control (Figure 4d, top panel) or anti-TGFβ antibody (Figure 4d, bottom panel) as described in the Materials and Method section. As indicated by Von Kossa staining, upon treatment with anti-TGFβ antibody, mineralized matrix production in primary calverial osteoblasts was increased by approximately 2-fold, when compared with isotype control antibody (Figure 4e, e; p = 0.005). This suggests anti-TGFβ antibody directly increases osteoblast differentiation, a parameter likely to contribute to the overall increase in bone mass.

### Anti-TGFβ antibody treatment increases mineral-to-collagen ratio in tumor-bearing animals

Confocal Raman Spectroscopy revealed that inhibiting TGFβ signaling with 1D11 increased the mineral-to-collagen ratio in the metaphyseal cortex of the tibia (Table 2). Of note, 1D11 did not affect the Type-B carbonate substitution, a measure of mineral distortion. Moreover, it did not affect crystallinity. This suggests that the suppression of TGFβ increased the rate of mineral accumulation in the organic matrix but not the structure of mineral crystals themselves. We anticipate this to be a reflection of improvement of osteoblastic activity with anti-TGFβ treatment.

**Table 2:** Suppression of TGFβ by anti-TGFβ antibody 1D11 increased the mineral-to-collagen ratio.

	13C4	1D11	% change	p-value
Mineral-to-collagen ratio	18.7±2.2	20.8±2.1	11.2	0.0287
Carbonate substitution	0.134±0.01	0.137±0.009	2.3	0.4417
Crystallinity	0.0463±0.0005	0.0461±0.0005	−0.3	0.5623

Confocal raman spectroscopy was performed on mice bearing MDA-MB-231 tumors in bone treated for 4 weeks with 1D11 or 13C4 antibodies as described in materials and methods section. At least nine spectra were analyzed per specimen and the mean mineral-to-collagen ratio, Type-B carbonate substitution, and crystallinity were scored. Both mineral-to-collagen ratio and carbonate substitution increased significantly upon 1D11 treatments compared to control. Mean ± standard deviation is shown, p value was determined using Wilcoxon test.

## Discussion

Despite major advancement in the treatment and prevention of early stage breast cancers, a large number of patients remain at risk of developing painful osteolytic bone metastases [3]. Although current anti-resorptive therapies using bisphosphonates are successful in preventing further bone resorption, they cannot repair the previously damaged bone. This leaves the patients with a high risk of pathological fracture and an increased morbidity and mortality. Thus, there is an urgent need for therapies directed at rescuing bone loss. Anti-TGFβ antibodies have been reported to reduce metastatic tumor burden related to breast cancers [31], [49]. Our data presented herein confirms these results in two preclinical breast cancers to bone metastases models and extends those to demonstrate that anti-TGFβ treatment increases mineralized matrix formation by osteoblasts as well as increases bone mass in preclinical bone metastasis models. Therapeutic approaches with a potential to maintain normal osteoblast activities and reducing osteoclastic bone resorption represent a novel paradigm.

While several laboratories have tested the efficacy of the anti-TGFβ antibody against inhibition of tumor burden, much less has been reported on the possible efficacy of this agent toward new bone formation. In addition to histology and microCT analysis, we have used cutting-edge biochemical techniques to analyze the composition of the bone in the tumor-bearing animals. We have previously reported that in normal murine bone, anti-TGFβ treatment increases the number of osteoblasts and decreases the number of osteoclasts, thereby increasing the overall bone mass [35]. In agreement with this, similar findings were noted by other groups using a small-molecule inhibitor of TGFβ receptor kinase, SD208, suggesting that blocking excess TGFβ is overall beneficial to the bone [33], [50]. Although the number of osteoblasts and osteoclasts is critical in maintaining bone remodeling, healthy bone formation also depends on the normal functioning of these cell types. The amount of bone is not always the exact measure of whether bone tissue is healthy and capable of normal load bearing involved in everyday activities. Of note, patients suffering from osteolytic bone damage often present with pathological fracture at the time of diagnosis. The suppression of TGFβ signaling affected tissue-level properties, namely bone resorption and mineralization. By studying TGFβ1 transgenic mice, Balooch et al. [16] found that decrease in tissue modulus is a function of increased TGFβ signaling. Likewise, treating young mice (4 weeks of age) with a pharmacological inhibitor of the TGFβ type I receptor (TβRI) kinase for 6 weeks increased both the degree of mineralization, as determined by X-ray tomography, and elastic modulus of the tibia cortex, as determined by nanoindentation [33]. Similarly, the current study using tumor-bearing animals found that TGFβ suppression increased the Raman-derived measure of mineralization (mineral-to-collagen ratio). This suggests that an anti-TGFβ antibody may prevent, if not reverse, the negative effect of tumors on bone quality.

Our finding using tumor-bearing animals has revealed that anti-TGFβ treatment modulated both osteoclast and osteoblast cell compartments, making this therapy more appealing for rescuing bone loss in osteolytic tumor models. We have demonstrated that anti-TGFβ antibody treatment inhibits osteoclast formation *in vivo*. In addition, a direct negative effect on osteoclast formation was demonstrated using bone marrow mononuclear cells. Of note, we have previously reported that in normal bone, there is almost 50% decrease in number of osteoclasts in 1D11-treated mice [35]. In addition to the direct effect on both osteoblasts and osteoclasts, osteoblast-mediated osteoclastogenesis was also inhibited using this approach. Much focus has been given to develop therapies directed to inhibition of osteoclastic bone resorption to prevent osteolytic bone damage. In osteolytic bone disease, osteoblast differentiation is often suppressed [51]. It has been reported that TGFβ modulates osteoblast differentiation [52]. Using an *ex vivo* assay, we have demonstrated that 1D11 antibody treatment increases mineralized matrix formation by calvarial osteoblasts, compared to the control antibody, which may likely contribute to an increase in the bone mass. In addition to make new bones, osteoblasts also maintain the homeostasis of osteoclast formation in the bone. Using a co-culture assay system, we have also demonstrated osteoblast-mediated osteoclastogenesis was inhibited by anti-TGFβ treatment. This emphasizes an indirect yet very important role for osteoblasts in affecting osteolytic bone damage. Data presented here exploits the concept of intervention of osteolytic bone damage by decreasing osteoclastic resorption and increasing osteoblastic differentiation simultaneously.

In agreement with previously reported anti-tumor efficacy of 1D11, we have also shown that tumor burden has been significantly reduced in both 4-week and 2-week treatment regimen (data not shown). As a possible mechanism of reduced tumor burden in the bone, 1D11 was able to inhibit TGFβ-mediated upregulation of Gli2 and PTHrP in MDA-MB-231 cells. Since the vicious cycle of bone metastasis is driven by multiple cell types in the bone [21], an effective therapy should target all these components to successfully cure the disease. We conclude that, in the two preclinical models used in this study, treatment with an anti-TGFβ antibody preserved bone volume and architecture, decreased tumor lesion number and size, and decreased osteoclast numbers. The overall effect of 1D11 appears to be partly on tumor cells and partly on the bone microenvironment, resulting in both improvement of bone volume and reduction in skeletal metastasis. We suggest that an approach to neutralize excess TGFβ might be a promising therapy for the treatment of patients with breast cancer metastasis to bone and may be successful in reducing bone related complications.
